# Prevalence and associated factors of circumcision among daughters of reproductive aged women in the Hababo Guduru District, Western Ethiopia: a cross-sectional study

**DOI:** 10.1186/s12905-016-0322-6

**Published:** 2016-07-22

**Authors:** Mulugeta Gajaa, Negash Wakgari, Yigzaw Kebede, Lemma Derseh

**Affiliations:** Department of Statistics, Addis Ababa Sciences and Technology University, Addis Ababa, Ethiopia; School of Nursing and Midwifery, College of Medicine and Health Sciences, Hawassa University, Hawassa, Ethiopia; Department of Biostatistics and Epidemiology, University of Gondar, Gondar, Ethiopia

**Keywords:** Circumcision, Daughter, Ethiopia, Hababo Guduru

## Abstract

**Background:**

Female genital mutilation is currently a public health problem which needs investigation and immediate action. Ethiopia is the second-ranked African country in terms of having higher numbers of circumcised girls. This study aimed to determine prevalence and associated factors of circumcision among daughters of reproductive aged women.

**Methods:**

A community based cross-sectional study was conducted on 610 mothers. The total sample was allocated proportionally in three randomly selected kebeles based on the number of reproductive age mothers with at least one daughter under 15 years old. A systematic random sampling technique was used to draw the respondents. A structured and interviewer administered questionnaire was used to collect data. Logistic regression analyses were used to see the association of different variables.

**Results:**

Out of 610 mothers, 293 (48 %) had at least one circumcised daughter. Having a good knowledge about genital mutilation (Adjusted Odds Ratio [AOR] =0. 14, 95 % CI: 0.09–0.23), positive attitude (AOR = 0. 26, 95 % CI: 0.16–0.43), being literate (AOR = 0.50, CI: 0.28–0.91) and living in urban area (AOR = 0.30, 95 % CI: 0.17–0.51) had a lower odds of female genital mutilation. In addition, not knowing genital mutilation as a crime (AOR = 5, 95 % CI: 3.07–8.19), and being in the age group of 40–49 (AOR = 2.56, 95 % CI: 1.40–4.69) had a higher odds of having circumcised daughter. Furthermore, fathers being traditional religion followers (AOR = 0.22, 95 % CI: 0.07–0.74) had less odds of having a circumcised daughter as compared to those who follow Ethiopian Orthodox Christian.

**Conclusions:**

In this study, about half of the mothers had at least one circumcised daughter. Mothers’ knowledge, attitude, age, residence, educational status and fathers’ religion were significantly associated with female genital mutilation. Hence, convincing mothers about the ill effects of circumcision and working with religious leaders is recommended.

## Background

World Health Organization (WHO) defines Female Genital Mutilation (FGM) as “all procedures that involve partial or total removal of the external female genitalia or other injury to the female organs for non-medical reasons [1]. Globally, prevalence of mothers who have one or more circumcised daughter was ranged from 3 % in Niger up to 73 % in Mali [2].

In Africa and Middle East countries, FGM remains a public health burden with its immediate and long term complications such as shock, sepsis, urine retention, tetanus, infertility, child birth complication and new born deaths [3, 4]. Studies revealed that knowledge, attitudes and perception about female genital mutilation in the communities and those of health care providers are big challenges in eliminating its practice in developing countries [1, 5–13].

Ethiopia is the second-ranked African country by the number of circumcised girls and women (23.8 million) next to Egypt [14]. In spite of the international communities’ commitment to make a zero tolerance of FGM [2], it is still being practiced in different ethnic groups of Ethiopia [8, 10, 14, 15]. The common justifications given for practicing FGM in Ethiopia include maintaining virginity, respecting the tradition, preventing social prejudice and religion related reasons [13–17]. The Ethiopian demographic and health survey conducted in 2005 shows that about half of the daughters had been circumcised before celebrating their first birthday [16]. Previous studies reported that a significant number of women with at least one daughter circumcised in Oromia regional state of Ethiopia [10, 17, 18]. Most of the studies conducted so far were focused on the older age group despite more than 95 % of circumcisions being conducted before 15 years of age [8, 17]. The decision for the daughter’s circumcision was made by their mothers due to the social expectation that the mother should take care of the daughters issue in various aspect [15]. For instance, daughters’ behavior such as sexual desire and relationship is closely observed by their mother and they intended to maintain FGM as a culture transferred from their mother [13]. Hence, this study provides information on female genital mutilation and associated factors of circumcision among daughters of reproductive aged women in the Hababo Guduru district, Western Ethiopia.

## Methods

### Study setting and population

The study was conducted from April 5 to 26, 2014 in the Hababo Guduru district, Horro Guduru Wallaga Zone of Oromia National Regional State, Western Ethiopia. Hababo Guduru district is 303 Kms away from Addis Ababa, the capital city of Ethiopia. There are 13 kebeles (a part of the district) in the study area. According to a 2007 Ethiopian national census report, the total populations of this district were 45,325 of which nearly half were females. With regard to religions, 42.15 % of the inhabitants were Ethiopian Orthodox Christians, 40.19 % were Protestants, and 16.93 % represented traditional beliefs [19]. According to information obtained from the Hababo Guduru health office in May, 2014, the total number of mothers in the district aged between 15–49 years was 9867 and the total numbers of girls under the age of 15 years was 12,893. All reproductive age women in the Hababo Guduru district, who had at least one daughter under 15 years old, were considered as source population. Those who have at least one daughter under 15 years old in the selected kebeles and who were available during the data collection period were considered as the study population.

### Study design, sample size, and sampling procedure

A community based cross-sectional study design was employed. Sample size was determined after calculating and comparing the sample sizes obtained for the study objectives. For the first objective, sample size was calculated by assuming, 95 % CI, 5 % margin of error and prevalence (23 %) of mothers who have at least one circumcised daughter under 15 years age [20]. Double population proportion formulae were used to address the second objective by taking the following assumptions into consideration: prevalence of mothers having circumcised daughters among uncircumcised mothers was 49.6 % with OR = 2 and prevalence of mothers having at least one circumcised daughter among knowledgeable mothers about the ill effects of FGM was 15 % with OR = 0.02 [21]. Epi-Info version 3.5.4 was used to calculate sample size when factors considered. Comparing sample size calculated for both specific objectives; the highest figure = 296 were obtained to address both specific objectives (Table 1). Hence, the total sample size with 2 design effect (since the selection was conducted in two stages: at kebele and household level), and 5 % non response rate became 622.

**Table 1:** Sample size determination

Sample size determination by using prevalence of mothers having circumcised daughters’ (outcome variable)		
Variables	Assumptions	*N*
Prevalence of mothers who had at least one circumcised daughter	*P* = 23 %, d = 0.05, CI = 95 %	$n=\frac{1.96^2*0.23*0.77}{0.05^2}=272$
Sample size determination considering factors		
Factors	Assumptions	n
Knowledge about ill effect of FGM	OR = 0.02, *P* = 15 %, Ratio 1:1, Power = 80 %, CI = 95 %	128
Mothers circumcision status	OR = 2, *P* = 49.6 %, Ratio 1:1, Power = 80 %, CI = 95 %	296

**Fig. 1:**
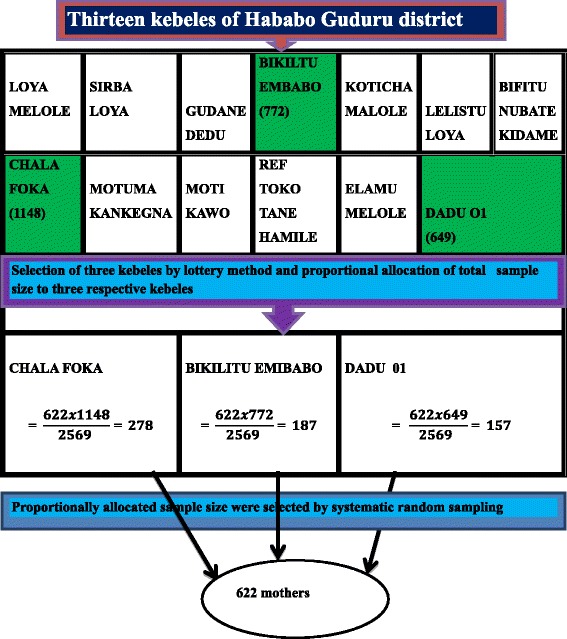
Schematic presesentation of sampling procedures

Two stage random sampling was undertaken to recruit study participants. Firstly, among a total of thirteen kebeles, three kebeles (Chala Foka, Bikiltu Embabo and Dadu kebele 01) were randomly selected. Following this, the total sample was allocated proportionally to each kebele based on the number of reproductive age mothers with at least one daughter under 15 years age. During the study period, there were 1148, 772 and 649 reproductive age mothers with at least one daughter under 15 years age in Chala Foka, Bikiltu Embabo and Dadu kebele 01, respectively. Accordingly, 278, 187, and 157 participants were invited from Chala Foka, Bikiltu Embabo and Dadu kebele 01, respectively. The respondents were drawn from the three randomly selected kebeles in the district by using a systematic random sampling method. Every 4th mother was selected and interviewed after identifying the first study participant by using the lottery method (Fig. 1). Mothers who had at least one circumcised daughter under 15 years old were asked required information about her recently circumcised daughter, which enabled us to minimize recall bias and assess the current practice.

### Data collection instruments

A structured and interviewer administered questionnaire was used to collect data. Relevant literature was reviewed to develop the tool and to include all the possible variables that address the objective of the study [5, 8–10, 13, 15, 17, 21, 22]. The questionnaire was first prepared in English and then translated into the local language (Afan Oromo), and back into English to maintain conceptual consistency. The questionnaire comprised different parts such as socio-demographic characteristics of respondents, knowledge about female genital mutilation, and their attitude towards FGM*.*

To check internal consistency of the tool, Cronbach’s alpha analysis was conducted with the value of 0.79, which implies that the items have high internal consistency. The instrument was pre-tested on 36 respondents in kebeles other than Bikiltu Embabo, Chala Foka and Dadu kebele 01. Findings from the pre-test were used to modify the instrument. Nine health extension workers and three BSc midwives were employed as data collectors and supervisors, respectively. All data collectors were females, to maximize response rate since mothers are more likely to talk freely about FGM with females than males. Before the actual work, both data collectors and supervisors were given one day training about the aim of the study, procedures, and data collection techniques by going through the questionnaires.

Knowledge about female genital mutilation was measured by using eight knowledge questions. In order to produce a more objective assessment of knowledge about circumcision a scoring method was devised and a knowledge score for each participant was obtained by adding up the score for correct response given to selected questions in the questionnaire. A score of mean value and above to knowledge related questions was considered as a good level of knowledge, while a score of less than the mean value indicates poor level of knowledge. Similarly, participants’attitude towards female genital mutilation was assessed by using a 3-point Likert scale as individuals responding agree for positive attitude was given scores of 2 and 0 for those who responded as disagree, while the above scores were reversed for negative attitude questions. There were four positive (discouraging FGM) statement assigned by (disagree = 0, neutral = 1, agree = 2) and four negative (encouraging FGM) statement assigned by (disagree = 2, neutral = 1, agree = 0) [14]. Finally, the total score was dichotomized into positive and negative attitude by taking the mean score as a cutoff point (Mean score or more = positive attitude and less than the mean score = negative attitude).

### Data processing and analysis

The collected questionnaire was checked manually for its completeness, coded and entered into Epi-Info version 3.5.4 statistical package, then exported to SPSS version 20.0 for further analysis. Descriptive and summary statistics were done. Both bivariate and multivariate logistic regression analysis was used to determine the association of each independent variable with the dependent variable. Variables significant in bi-variate analysis (*p*-value less than or equal to 0.2) were entered into a multivariate logistic regression model to adjust the effects of confounders on the outcome variable. Odds ratio with their 95 % confidence intervals were computed to identify the presence and strength of association, and statistical significance was declared if *p* < 0.05.

## Results

### Socio-demographic characteristics of study participants

A total of 610 mothers were included in the study (response rate = 98 %). The ages of respondents were between 17–49 years with a mean age of 32.2 (SD = 7.2). About 55.4 % of the respondents were between 26–35 years of age. Ninety one percent of the study participants were married and 75 % were rural residents. Both Orthodox and Protestant religions shared an almost equal number of participants 42 and 40.8 % respectively. The majority (74.6 %) of the study participants could not read and write. Only 4.3 % of the respondents were attend primary and above school. About 86.7 % of study participants were housewives and 35 % of the respondents had two daughters (Table 2).

**Table 2:** Socio demographic characteristics of mothers in the Hababo Guduru district, west Ethiopia, 2014

Variables	Frequency	Percentage
Marital status		
Married	555	91.0
Single	55	9.0
Age		
15–29	59	9.7
30–39	309	50.6
40–49	242	39.7
Number of daughters		
One	125	20.5
Two	214	35.1
Three	166	27.2
Four and above	105	17.2
Occupation		
House wife	529	86.7
Civil servant	19	3.1
Merchant	50	8.2
Student	12	2.0
Educational status		
Cannot read and write	455	74.6
Read and write	129	21.1
Primary and above	26	4.3
Religion		
Orthodox Christian	256	42.0
Protestant Christian	249	40.8
Traditional belief	105	17.2
Residence		
Rural	455	74.6
Income per month		
<555 birr	375	61.5
556–1233 birr	213	34.9
>1233 birr	22	3.6

### Prevalence of mothers with at least one circumcised daughter under 15 years of age

**Fig. 2:**
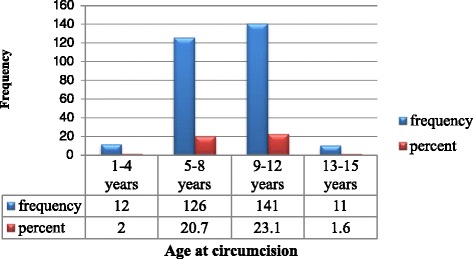
Age at circumcision among under 15 years old daughters in the Hababo Guduru district, west Ethiopia, 2014

Out of 610 mothers, 48 % had at least one circumcised daughter under the age of 15. About 98 % of study participants were circumcised. The modal age at which circumcision conducted was 5–12 years old which comprises about 92.4 % and the mean age at which circumcision conducted was 8.7 ± 2.3 SD (Fig. 2).

### Mother’s knowledge about female genital mutilation and the reasons stated for circumcising daughter

**Fig. 3:**
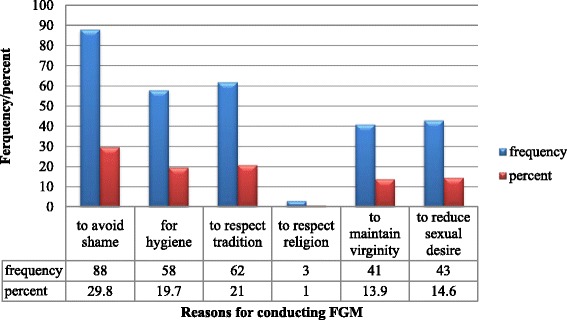
Reasons stated for circumcising daughters in the Hababo Guduru district, west Ethiopia, 2014

Two hundred eighty two (46.1 %) mothers were knowledgeable about the ill effects of FGM. Few numbers of mothers (6 %) knew that HIV could be transmitted by FGM (Table 3). The major reasons stated by respondents for circumcising their daughters were to avoid shame and to respect tradition which accounted for 88 (29.1 %), and 62 (21 %) respectively (Fig. 3).

**Table 3:** Knowledge of mothers about genital mutilation in the Hababo Guduru district, west Ethiopia, 2014

Knowledge about FGM	Yes	%
Does FGM represent a health problem?	62	10.2
Does FGM have any categories?	14	2.3
Can FGM bring infection?	36	5.9
Can HIV transmitted by FGM?	37	6.0
Does FGM violate human right?	9	1.5
Does FGM affect both mother and child during birth?	18	2.8
Does religion order circumcision of daughters?	47	7.7
Does Ethiopian law criminalize circumcising daughter?	59	9.7

### Attitude of mothers towards female genital mutilation

Nearly half, 287 (47 %) of the participants in this study had a negative attitude towards FGM. Most of the respondents, 592 (97.1 %) agreed that being left uncircumcised is shameful and 530 (86.9 %) agreed that an uncircumcised daughter may not get married. More than half, 352 (57.7 %) of mothers agreed that uncircumcised women do not respect other individuals. About half, 310 (50.8 %) agreed that FGM did not prevent daughters from breaking utensils. A lower proportion, 231 (37.9 %) of mothers agreed that circumcision preserves virginity. Nearly two thirds, 421 (69 %) of the respondents disagreed with the ideas of FGM as a harmful traditional practice. In addition, 525 (86.1 %) disagreed with the statement that FGM is not good for hygiene (Table 4).

**Table 4:** Attitude of mothers towards genital mutilation in Hababo Guduru district, west Ethiopia, 2014

Attitude towards FGM	Disagree		Neutral		Agree	
	Frequency	%	Frequency	%	Frequency	%
FGM is a harmful traditional practice which should be stopped	421	69	8	1.3	181	29.7
FGM would not prevent daughters from breaking utensils	297	48.7	3	0.5	310	50.8
Uncircumcised daughters are not isolated	357	58.5	14	2.3	239	39.2
FGM is not good for hygiene	525	86.1	2	0.3	83	13.6
Nobody needs to marry uncircumcised women	75	12.3	5	0.8	530	86.9
Being left without circumcision is shame	18	2.9	0	0	592	97.1
Uncircumcised women do not respect other individuals	251	41.2	7	1.1	352	57.7
Circumcision preserves virginity	375	61.5	4	0.6	231	37.9
Over all attitude of respondents towards FGM	Positive				323	53
	Negative				287	47

### Factors associated with having circumcised daughters

In bivariate analysis except circumcision status of mother, income, marital status of the mother and the marital status of a father all were found to be associated with having circumcised daughter. In multivariable analysis all fathers’ socio-demographic characteristics were insignificant except father’s religion. However, mothers’ age, residence, knowledge, attitude, educational status, and knowing FGM as a crime were found to be significantly associated with having circumcised daughter. Mothers who had a good knowledge (AOR = 0.14, 95 % CI: 0.09–0.23) and positive attitude (AOR = 0.26, 95 % CI: 0.16–0.43) were 86 % and 74 % less likely to have circumcised daughters as compared to their counterparts, respectively. Those who were living in urban area (AOR = 0.30, 95 % CI: 0.17–0.51) were 70 % less likely to have circumcised daughters as compared to living in a rural area. Similarly, those who were literate (AOR = 0.50, CI: 0.28–0.91) were 50 % less likely to have circumcised daughters as compared to those who were illiterate. In addition, not knowing circumcision as a crime (AOR = 5, 95 % CI: 3.07–8.19), and being in the age group of 40–49 (AOR = 2.56, 95 % CI: 1.40–4.69) represented a 5 and 2.56 times more likely to have circumcised daughters as compared to those who know circumcision as a crime and in the age group of 15–29, respectively. Furthermore, fathers who followed traditional religion (AOR = 0.22, CI: 0.07–0.74) were 78 % less likely to have circumcised daughters as compared to those who followed an Orthodox religion (Table 5).

**Table 5:** Factors associated with having one or more daughter circumcised in the Hababo Guduru district, west Ethiopia, 2014

Variables			Have circumcised Daughters		
	Yes	%	COR (95 % CI)	AOR (95 % CI)	*P*- value
Marital status^a^					0.43
Married	260	88.7	1	1	
Singles	33	11.3	1.70 (0.97–3.00)	1.36 (0.64–2.89)	
Age^a^					0.005*
15–29	22	7.5	1	1	
30–39	129	44	1.82 (1.26–2.63)	1.95 (1.15–3.31)	
40–49	142	48.5	2.84 (1.84–4.40)	2.56 (1.40–4.69)	
Residence^a^					<0.001*
Rural	245	83.6	1	1	
Urban	48	16.4	0.39 (0.26–0.57)	0.30 (0.17–0.51)	
Religion^a^					0.07
Orthodox Christian	125	42.7	1	1	
Protestant Christian	104	35.5	0.75 (0.53–1.10)	0.98 (0.46–2.09)	
Traditional belief	64	21.8	1.64 (1.03–2.60)	3.86 (1.14–13.07)	
Education^a^					0.04*
Illiterate	241	82.3	1	1	
Literate	46	15.7	0.49 (0.33–0.74)	0.50 (0.28–0.91)	
Primary and above	6	2	0.27 (0.11,0.67)	0.42 (0.12–1.42)	
Occupation^a^					0.64
Housewife	264	90.1	1	1	
Student	4	1.4	0.50 (0.15–1.69)	2.19 (0.27–18.24)	
Merchant	22	7.5	0.80 (0.44–1.41)	1.71 (0.65–4.52)	
Civil servant	3	1	0.19 (0.05–0.65)	0.80 (0.15–4.30)	
Knowledge^a^					<0.001*
Poor knowledge	229	78.2	1	1	
Good Knowledge	64	21.8	0.13 (0.09–0.18)	0.14 (0.09–0.23)	
Attitude^a^					<0.001*
Negative attitude	251	85.7	1	1	
Positive attitude	42	14.3	0.12 (0.08–0.18)	0.26 (0.16–0.43)	
Income^a^					0.23
<555birr	187	63.8	1	1	
556-1233birr	101	34.5	0.91 (0.65–1.27)	0.91 (0.55–1.51)	
>1233birr	5	1.7	0.30 (0.11–0.82)	0.24 (0.05–1.24)	
Know FGM as criminal^a^					<0.001*
Yes	131	44.7	1	1	
No	162	55.3	4.45 (3.13–6.32)	5.00 (3.07–8.19)	
Religion of father					0.04*
Orthodox Christian	112	43.0	1	1	
Protestant Christian	96	36.9	0.69 (0.47–0.99)	0.62 (0.29–1.34)	
Traditional belief	52	0.2	1.21 (0.75–2.00)	0.22 (0.07–0.74)	

*Significant variables (*P* ≤ 0.05)

^a^Information related to mother, 1 = reference

## Discussion

This study attempted to determine the prevalence and associated factors of circumcision among daughters of reproductive aged women in the Hababo Guduru district, Western Ethiopia. In this study, 48 % of the study participants had at least one circumcised daughter. This finding is lower than the studies conducted in Upper Egypt (77.3 %) [23] and East Gojjam Zone, Ethiopia (62.7 %) [21]. However, it is higher than the findings of national reports of Ethiopia (38 %) and the report for Oromia region (34.9 %) [14]. These differences might be due to differences in place of the studies that might be explained by different strategies in combating FGM and different attitude level of the respondents about FGM. The other possible explanation could be the difference in cultural background of the study participants [21].

In the present study, the common reasons given for their daughter’s circumcision were to avoid shame, to prevent breach of traditional and religious respect, to maintain virginity, to keep the hygienic status in the vulva area and to reduce sexual desire, which is in agreement with the study conducted in Hadiya zone, Southern Ethiopia [8]. Most of the circumcisions (92.8 %) in the current study were performed by traditional circumcisers, which is also in line with the UNICEF’s report [2]. Most (98.2 %) of the mothers themselves reported they had undergone FGM. This is similar to the WHO report of Somalia (98 %) [24]. However, the current finding is higher than the study done in Amhara region, Ethiopia (84 %) [21]. This variation might be due to the high prevalence of younger mothers in the previous study than the current study. For instance, all 11 (1.8 %) mothers ‘who were not circumcised in this study were below 20 years old.

Knowledge about health ill effects of FGM was significantly associated with the daughters’ circumcision status. Mothers who had a good level of knowledge about FGM had 86 % less odds of having circumcised daughters as compared to those who had a poor level of knowledge. This study is comparable with study done in Amhara region [21]. Furthermore, the study found attitude as another factor influencing FGM. Female genital mutilation was significantly higher among mothers who had a negative attitude as compared to those who had a positive attitude. This is in line with the studies done in Egypt and Somali [22, 23].

Mothers’ circumcision status was insignificantly associated with daughters’ circumcision status, but in other studies circumcised mothers were more likely to have circumcised daughters as compared to their counterparts [21, 25]. The possible explanation for this difference might be due to small number of uncircumcised mothers included in the current study. In addition to this, mothers who were circumcised might be more knowledgeable about the ill health effects of FGM as compared to their counterparts [26], since they have experienced the problem of undergoing FGM, they may avoid the procedure for their daughters. Fear of isolation and insult that non circumcised daughters faced may enforce them to circumcise their daughters and fear of health consequence that the circumcised mothers faced restrict them from circumcising their daughters; these two things might nullify the effects of mother’s circumcision status on having daughters circumcised.

In the current study, there is a significant association between educational status of mothers and having daughters circumcised. This study finding is comparable with several studies conducted [25–28]. Age of mothers is one of the factors determining to have circumcised daughters. Those mothers who were in the age group of 30–39 and 40–49 had 1.95 and 2.56 times greater odds of having circumcised daughters than those who were in the age group of 15–29. This might be explained by young mothers are closer to information and FGM is being discouraged. This is in line with the study done in the Upper Egypt [23]. Residence of mothers also significantly affects the daughters’ circumcision status. Where mothers reside in urban areas they were 70 % less likely to have circumcised daughters as compared to their counterparts. The current study is consistent with the study conducted in Nigeria [28]. Mothers who did not know FGM as crime were 5 times more likely to have circumcised daughters as compared to their counterparts. This is similar with the study conducted in Amhara region, Ethiopia [25]. Religion of father is another factor influencing daughters’ circumcision. This finding is consistent with the study conducted in Upper Egypt [23].

### Limitations

Response, recall and social desirability bias are the potential limitations of this study. However, numerous scientific procedures have been employed to minimize the possible effects. For instance, to reduce the response bias and to obtain a genuine response a discussion was held with the respondents about the aim of the study. Moreover, procedures such as supervision, pretest of data collection tools, and adequate training of data collectors and supervisors were carried out.

## Conclusions

Prevalence of mothers who have one or more daughter circumcised was found to be high as compared to national and regional level. Mothers’ knowledge, attitude, educational status, residence, age, knowing FGM as a crime and religion of fathers were significantly associated with having at least one circumcised daughter. Giving attention to mothers living in rural areas, taking necessary measurement on traditional circumcisers, working with religious leaders, alerting the community that FGM is a crime and giving awareness about the ill effects of FGM is strongly recommended. District health office should work with community to eliminate traditional belief of being shamed and unhygienic if uncircumcised.

## Abbreviation

FGM, female genital mutilation
